# Contribution of Single-Cell Transcriptomics to the Characterization of Human Spermatogonial Stem Cells: Toward an Application in Male Fertility Regenerative Medicine?

**DOI:** 10.3390/ijms20225773

**Published:** 2019-11-16

**Authors:** Anne-Sophie Gille, Clémentine Lapoujade, Jean-Philippe Wolf, Pierre Fouchet, Virginie Barraud-Lange

**Affiliations:** 1 UMRE008 Stabilité Génétique, Cellules Souches et Radiations, Laboratoire des Cellules Souches Germinales, IRCM, Université de Paris, Université Paris-Saclay, CEA, F-92260 Fontenay-aux-Roses, France; clementine.lapoujade@cea.fr (C.L.); pierre.fouchet@cea.fr (P.F.); 2 Team Genomic Epigenetic and Physiopathology of Reproduction, Department of Genetic, Development and Cancer, Cochin Institute, Inserm U1016, 22 rue Méchain, 75014 Paris, France; jean-philippe.wolf@aphp.fr (J.-P.W.); virginie.barraud-lange@aphp.fr (V.B.-L.); 3 Sorbonne Paris Cité, Faculty of Medicine, University Paris Descartes, Assistance Publique-Hôpitaux de Paris, University Hospital Paris Centre, CHU Cochin, Laboratory of Histology Embryology Biology of Reproduction, 123 boulevard de Port Royal, 75014 Paris, France

**Keywords:** single-cell, transcriptomic, spermatogonial stem cells, human, fertility preservation, regenerative medicine

## Abstract

Ongoing progress in genomic technologies offers exciting tools that can help to resolve transcriptome and genome-wide DNA modifications at single-cell resolution. These methods can be used to characterize individual cells within complex tissue organizations and to highlight various molecular interactions. Here, we will discuss recent advances in the definition of spermatogonial stem cells (SSC) and their progenitors in humans using the single-cell transcriptome sequencing (scRNAseq) approach. Exploration of gene expression patterns allows one to investigate stem cell heterogeneity. It leads to tracing the spermatogenic developmental process and its underlying biology, which is highly influenced by the microenvironment. scRNAseq already represents a new diagnostic tool for the personalized investigation of male infertility. One may hope that a better understanding of SSC biology could facilitate the use of these cells in the context of fertility preservation of prepubertal children, as a key component of regenerative medicine.

## 1. Introduction

Recent advances in genomic technology offer the opportunity to sequence at single-cell resolution. Untargeted genome expression profiling of individual cells instead of discrete cell subpopulations within a tissue has revolutionized our comprehension of differentiation processes by offering a wealth of information that captures the identities of individual cells and characterizes their molecular properties under native conditions. This has been a breakthrough in the fundamental understanding of intrinsic cellular diversity and dynamics. Therefore, this new approach unmasks key questions about the heterogeneity and diversity of stem cells in developmental and cancer research that bulk analyses were unable to address. Among these single-cell technologies, explorative single-cell transcriptome sequencing (scRNAseq) produces a “molecular atlas” of gene expression. It helps to comprehensively catalogue the repertoire of cell types present in a complex biological system, to infer differentiation trajectories, and to describe state transitions. For example, scRNAseq has proven to be an effective means of establishing an impartial stocktaking of heterogeneous cell populations in various tissues including spleen [1], pancreas [2], lung [3], regions of the brain [4,5], and even in early embryonic development [6].

In addition to the many scRNAseq studies on spermatogenesis in neonatal, prepubertal, and adult mice [7,8,9,10,11,12,13,14,15,16,17,18], which provided unbiased classifications of spermatogonial stem cell (SSC) pools and differentiation states, an atlas of human spermatogenesis at three different developmental stages (neonatal, prepubertal, and adult) has also been described recently thanks to this method [12,19,20,21,22,23]. The atlas reveals the striking heterogeneity among testicular somatic and germinal cell populations and assesses the identity of human SSC (hSSC) and spermatogonial progenitors.

## 2. Spermatogenesis and the Spermatogonial Stem Cell Model

Spermatogenesis requires coordination of mitosis, meiosis, and spermiogenesis, the process through which diploid spermatogonia turn to haploid spermatids and then mature into sperm. Although spermatogenesis is highly ordered, it remains difficult to define. Histological studies have shown that changes in the cellular composition of the seminiferous epithelium occur in a wave-like fashion. Indeed, sets of germ cells at different stages of development can be seen along the seminiferous tubule, which is paved with structurally overlapping clonal units. This spatial and temporal asynchrony guarantees the continuous production of sperm [24]. Referred to as the “seminiferous epithelium cycle,” this particular architecture includes 12 stages in mice and 6 in humans [25,26].

Throughout the reproductive life of mammals, differentiation of a small pool of cells that are located on the basal membrane of the seminiferous tubules, the SSC, occurs. These cells can self-renew or differentiate, maintaining tissue homeostasis and ongoing spermatogenesis. The presence of stem cells in testes was clearly demonstrated by recovery of spermatogenesis after transplantation of donor testicular cells into murine germ cell-depleted testes [27,28]. Thus, testicular transplantation of SSC has indeed been found to lead to the efficient production of functional sperm and the restoration of fertility in several animal models [29,30,31,32,33], including non-human primates [34]. Furthermore, the exhaustion of the SSC population may be associated with defects in spermatogenesis, including the most severe infertility issue, Sertoli cell-only syndrome, in which there is complete germ cell aplasia.

In mice, the best characterized mammalian model, approximately 3000 to 4000 SSC (also called A_single_ (A_s_) spermatogonia) are found in the adult testis [25,35]. A_s_ spermatogonia can self-renew or differentiate into committed paired cells (A_p_) and then into chains of 4, 8, and 16 aligned (A_al_) spermatogonia after successive divisions, which are collectively called undifferentiated spermatogonia. These cells in turn differentiate into A1, A2, A3, A4, intermediate (In), and B spermatogonial cells, which actively proliferate, amplifying the number of differentiated spermatogonia, before entering meiosis [36]. Lineage tracing experiments using transgenic mice models to track various markers such as ID4, Pax7, Bmi-1 and GFRα1 showed the presence of mouse SSC (mSSC) in single A_s_ or short chains of spermatogonia [37,38,39,40]. Furthermore, some combinations of functional or cell surface phenotypic markers have been identified, making it possible to purify highly enriched fractions of mSSC, and it has been confirmed using transplantation assays that the regenerative potential of mSSC is found in the undifferentiated spermatogonial population [41,42,43,44,45]. Recently, an A_s_ hierarchical model has been refined. Stem cell activity is notably restricted to A_s_ that strongly express an ID4-EGFP transgene in a mouse model. The ID4-EGFP^bright^ population coexists with other A_s_ cells that express the transgene at a lower level, and this diminution is linked to a reduced stemness potential [46]. However, this model has been questioned, and Hara et al. introduced a new and attractive model for a stochastic regulation of mSSC that considers that spermatogonia expressing the receptor GFRα1 constitute an equipotent stem cell population in terms of regenerative potential, regardless of their A_s_ or syncytial unit status [40]. On another note, the importance of the spermatogonial specialized microenvironment to self-renewal has been shown. Named “the niche,” this microenvironment is composed of a set of supporting cells that includes Sertoli cells (SC), peritubular myoid cells (PMC), Leydig cells (LC), and blood and endothelial cells [47] and emits regulating signals such as glial cell line–derived neurotrophic factor (GDNF) and members of the fibroblast growth factor family (FGF) [48,49,50].

Compared to mice, the identification of the stem cell pool that gives rise to the spermatogenic lineage and of the molecular mechanisms that govern self-renewal and differentiation in humans remains largely elusive. The prevailing model is that human spermatogenesis arises from A_dark_ and A_pale_ spermatogonia, which are considered to represent reserve and active stem cells, respectively [51,52,53]. As defined in Clermont’s histological work, A_dark_ spermatogonia constitute a stock population of quiescent stem cells, while A_pale_ spermatogonia represent proliferating stem cells that self-renew and are primed to differentiate in B spermatogonia. In addition, the amplification phase of the differentiated spermatogonial progenitors in primates seems less important than in the mouse model [54]. Hence, simple extrapolation of the murine model to the human model is difficult, and significant efforts are needed to provide new insights that can lead to a better understanding of the physiology of hSSC. Reliable phenotypic markers that make it possible to purify and study individual populations of stem cells and progenitors are still lacking, and this is a major roadblock to our understanding of the self-renewal and regenerative potential of hSSC [55].

scRNAseq provides various types of information that may help establish an unbiased atlas of germinal and somatic cells in the human testis. A comprehensive analysis of gene expression in human testes could lead to the phenotypic characterization of hSSC and the identification of the key molecular factors and developmental trajectories that govern human spermatogenesis.

## 3. From Plate-Based Methods to Microfluidic Chips: A Wide Range of scRNAseq Techniques

**Figure 1:**
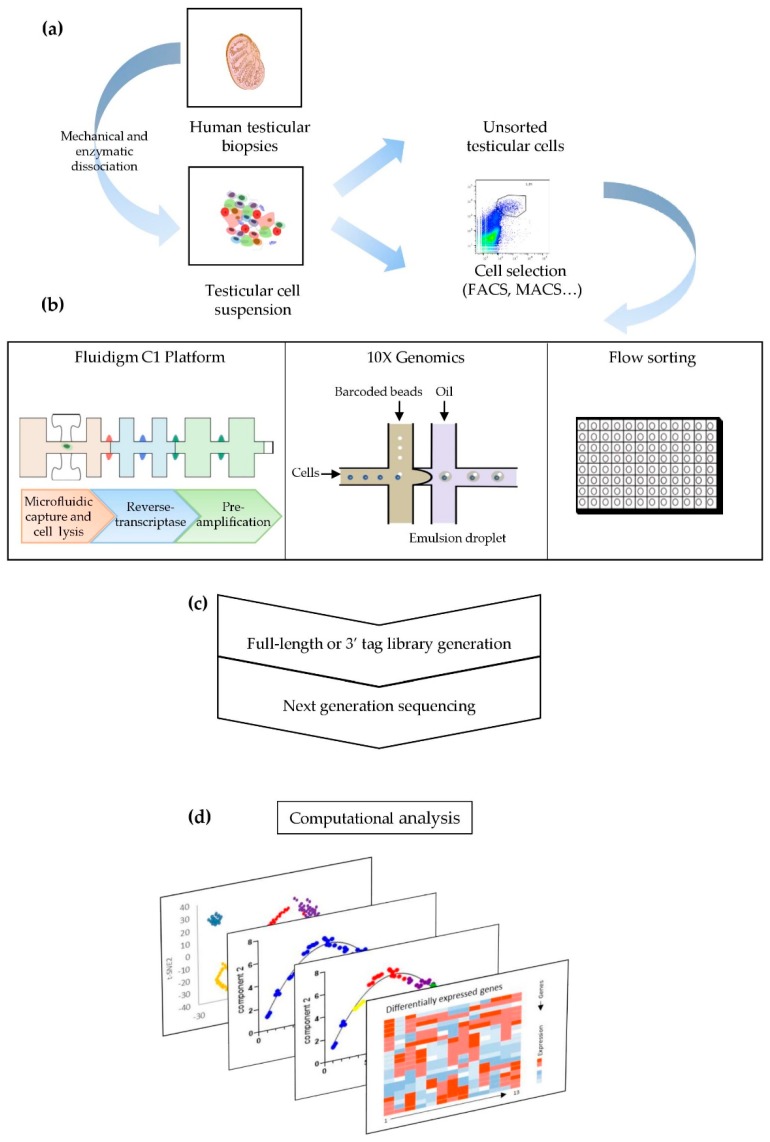
Single-cell transcriptome sequencing (scRNAseq) flowchart for the exploration of human spermatogenesis. (**a**) The testicular tissue is mechanically and enzymatically dissociated to get a cell suspension. The cells can either be sorted to focus on a specific cell population or directly screened for an unbiased analysis. (**b**) scRNAseq library is generated, using platforms as Fluidigm C1 or 10× genomics, for example, via successive steps of single cell isolation, cell lysis, reverse transcription of RNA into cDNA, followed by amplification. (**c**) Next generation sequencing—Smart-seq protocol provides full-length transcriptome analyses (e.g., in C1 platform), while Droplet-sequencing approaches generate 3′ tag RNAseq libraries. (**d**) The raw data are computationally analyzed and structured into clusters, developmental trajectories by pseudotime analysis and networks to characterize cell subsets.

RNA sequencing consists of the comprehensive screening of gene expression. It goes one stage further than genome exploration by considering huge variations in expression levels, which often involve variable splicing and resulting isoforms [56]. scRNAseq integrates messenger RNA (mRNA) but also long non-coding RNA (lncRNA) that play crucial roles in regulation [4]. Tang et al. were the first to describe a single-cell digital gene expression profiling assay performed on a single mouse blastomere [57] based on a cDNA amplification protocol previously used for microarray analyses [58] that was adapted for mRNAseq. Ten years later, many methods and automated instrumentations have been developed to obtain the transcriptome of an individual cell. The process requires the isolation of single cells and the preparation of an scRNAseq library, including cell lysis, capture and conversion of mRNA into cDNA and amplification of cDNA, followed by sequencing and computational analyses (Figure 1). Diverse scRNAseq protocols generate sequencing libraries with varying sensitivity and uniformity in their coverage of gene expression and must be selected according to the number of cells that is required and can be screened, their throughput, and economics.

Although single cells can be isolated by laser capture microdissection from solid specimens, micromanipulation or manual cell picking from a cell tissue suspension [59], cell segregation is usually performed via fluorescence-activated cell sorting (FACS) or microfluidics (Fluidigm C1 Platform, droplet-based-scRNAseq methods) [60]. However, it should be noted that isolation using antibodies may bias the analysis because only certain cells are selected for further individual screening [61].

Various protocols, each with its own advantages and weaknesses, have been proposed over the past decade. Microfluidic chips such as those developed by Fluidigm (C1 platform) outperform plate based methods by offering higher throughput; up to 800 cells can be automatically analyzed in one experiment, with the single-cell repartitioning being controlled by microscopy [62]. Several tools for constructing cDNA libraries from low-input RNA have been developed; one example is Smart-seq2 [63], a full-length method that allows efficient single-cell transcriptome sequencing with high sensitivity as it detects the expression of most genes of a cell [64].

The development of microdroplet-based methods represents a major breakthrough. In these methods, each cell is encapsulated in a nanolitre emulsion droplet containing barcoded bead-bound poly(dT) oligonucleotides [65,66]. The association of all RNA from one cell with a distinct barcode allows the investigator to track each transcript from an individual cell and consequently to perform massive parallel analysis of pooled cDNA libraries. Drop-Seq or InDrop-Seq techniques and the commercial Chromium™ platform from 10× Genomics^®^, which use this strategy, offer cost-efficient analyses of large numbers of cells (up to 10,000) per experiment [60].

New protocols are still emerging. Among them, Seq-Well does not require the capture of cells in droplets. Instead, single cells are driven by gravity into the picowells of a chip in which the size of the wells only permits the incorporation of a single barcoded bead plus an individual cell [67].

Another important technical implementation is the incorporation of unique molecular identifiers (UMIs) for cDNA library synthesis that enable better quantification of the number of scRNAseq reads. Because each transcript is identified by a unique UMI, this method makes it possible to distinguish read counts stemming from each reversed-transcribed transcript from false positive duplicates synthesized during cDNA amplification [68]. Since then, UMIs have been implemented in many scRNAseq methods, including drop-based (Drop-Seq, InDrop, and Chromium™ platforms) and well-based protocols (for example, scRNA-barcoding and sequencing, mcSCRB-Seq) [69].

In brief, Smart-seq2-based methods are effective tools for a better coverage of transcriptome, while the use of UMIs in other methods such as Drop-Seq makes it possible to obtain better quantification of RNA. Thus, micro-droplet-based methods utilizing UMIs and cell barcodes are advised when large numbers of cells must be analyzed, and Smart seq2-based methods, such as the method offered by the C1 platform, are adapted when small numbers of selected cells are examined.

## 4. Pre-Processing System and scRNAseq Data Computational Analysis

High-throughput scRNAseq provides a massive amount of data that must be interpreted using sophisticated computational tools. The major challenge is to develop a scRNAseq processing method that dissects complex information in a way to permit a proper understanding of transcriptome dynamics. Data processing of raw reads comprises the following steps: quality control (QC) of reads and filtering to eliminate low-quality sequences, dead cells, and multiplets resulting from the capture of several cells; demultiplexing based on the cell barcode assignments; alignment of the sequence to a reference database; and normalization and quantification (for UMIs or spike in transcripts) [70,71]. The expression profile is then structured into clusters, pseudotime analyses, or networks to characterize cell subsets.

First, appropriate algorithms such as principal component analysis (PCA) or t-distributed stochastic neighbor embedding (t-SNE), for example, project data onto a lower-dimensional object, simplifying the visualization of the results in the form of 2D or 3D objects. Clustering methods for scRNAseq data (hierarchical, k-means, or graph-based, among others) support discrete analyses that can detect distinct cell types or even rare cell subpopulations. The latter can be further characterized according to their differentially expressed genes. Open-source software tools such as Seurat support this type of analysis [72].

Single-cell trajectory inference is used to study cellular dynamics and to delineate transitions between different cell states in the differentiation process. ScRNAseq data are analyzed according to the concept of “pseudotemporal analysis” using, for example, Monocle software [73]. Cells are computationally ordered based on the similarities of their transcriptomes along a pseudotime axis from the beginning to the end of the differentiation process. Several methods that make it possible to draw complex trajectories from RNAseq data using pseudotime modelization have been developed [74]. The latest software updates (RNA velocity, Monocle 3) support modelling trajectories in which cells may cycle through recurrent intermediate states before reaching an endpoint of differentiation. Moreover, the open-source software single-cell trajectories reconstruction, exploration and mapping (STREAM) makes it possible to go even further in the inference of developmental trajectories by combining both epigenetic and transcriptomic data [7].

## 5. Limitations and Potential Improvements

The intrinsic characteristics of single-cell sequencing are accompanied by analytical and computational challenges. Transcriptome datasets may vary considerably due to a variety of biological factors on the one hand and, on the other hand, to technical parameters that differ between experiments and according to the scRNAseq methods used [62]. These elements should be critically considered when designing and evaluating such studies. Once processed and carefully interpreted, scRNAseq results should be validated at the RNA (RNA in situ hybridization, RT-PCR) and protein levels. Investigators must remain vigilant throughout the process because it suffers from limitations, especially when rare subsets of cells are analyzed.

First, discrepancies between biological replicates impact the robustness and reliability of the data. Variability among samples in response to treatments may result in batch effects, and the methods (enzymatic digestion, mechanical dissection) used to isolate cells may impact their transcriptional profiles [62,75]. Operating with multiple biological replicates for each condition is recommended to reduce batch effects [76].

Furthermore, because the number of cells that are ultimately profiled is critical for the capture and detection of rare cell types, the study of scarce populations may require preselection of cells. On another scale, tiny starting amounts of one cell’s transcripts could induce technical noise through disparities in amplification and heterogeneity in gene dropout (false negative) events [77,78]. Genes that are expressed at low levels are unlikely to be quantified using the currently available high-throughput scRNAseq methods, preventing them from being considered in cell-to-cell comparisons [79].

Technical variables such as the sequencing depth and the multiplet rate must be considered. For example, sequencing depths may vary widely between protocols, from 10,000 reads to 100,000 reads per cell in droplet-based scRNAseq studies to close to 1,000,000 reads per cell in Smart-seq-based studies [79]. Therefore, alternative transcripts are hardly detectable in high-throughput 3′ tag sequencing approaches. The 5′ ends of mRNA transcripts, which contain crucial information on gene regulation, are also difficult to define. The development of libraries targeting the 5′ ends of transcripts could help compensate for this lack of information [80]. In addition, high-throughput scRNAseq analyses exclude non-polyadenylated RNA such as some lncRNA and various non-coding RNA with regulatory functions such as microRNA (miRNA) and piwi-interacting RNA (piRNA) [81]. A promising strategy, single-molecule Nanopore direct RNA sequencing, uses a nanopore-based platform that eliminates the amplification stage, reducing the time required for library preparation and making it possible to investigate the whole transcriptome, including non-polyadenylated RNA [82]. The development of scRNAseq methods that use this approach will undoubtedly contribute to the production of a vast atlas of information on single-cell gene expression during the process of human spermatogenesis in the coming years.

scRNAseq captures the gene expression profiles of selected cells at a specific time point as they exist in vivo, but this expression profile is devoid of any temporal or spatial context. Different snapshots of individual cells from a clone and a holistic view of the clones should be methodically constructed according to the lineage timeline but must also be redesigned, as spatial location is a determinant of cell function and fate [48]. In the case of testicular tissue, arranging cells ranging from immature progenitors to sperm cells along pseudotime via their transcriptional profiles involves certain assumptions and imposes some limitations. It must be kept in mind that, if differentiation is a continuous process, cells differentiate asynchronously, and an scRNAseq experiment provides a one-time expression profile along the cells’ differentiation routes. Asynchronous differentiation of the different SSC clones in testis described by Kanatsu-Shinoahara et al. [83] and co-occurrence of different seminiferous epithelium cycles that involve somatic and germinal cell lineages raise the question of setting a trajectory (SSC to endpoint) for a given clone among others that are quiescent or at another differentiation stage.

The use of in situ hybridization (ISH) targeted approaches was first suggested to remedy the absence of spatial information and to complement the scRNAseq dataset. RNA can be localized in their native environment thanks to the use of complementary fluorophore-labelled probes [84]. Thus, single-molecule RNA fluorescence in situ hybridization (smFISH) offers the possibility to quantify and localize the expression of few genes within a tissue by using a multiplicity of probes targeting a single mRNA molecule. Sequential FISH methods such as sequential hybridizations to targeted RNA (seqFISH) [85] or multiplexed error-robust FISH (MERFISH) [86] (that detects the readout sequences in successive hybridization rounds) allow us to increase the number of the different RNA species (from 100 genes to 10,000 genes) that can be simultaneously quantified [87,88,89,90]. While fluorescence in situ sequencing (FISSEQ) or alternative methods have been proposed to analyze tissue with a higher throughput [91,92], other explorative methods have been also developed based on the capture of mRNA from a cell or tissue on glass slides coated with oligonucleotides associated with spatial barcodes, followed by library preparation and the massive throughput RNAseq [93,94,95].

Last but not least, the incompatibility of the scRNAseq approach with functional assays such as stem cell transplantation, lineage tracing, and tests of in vitro colony-forming capacity is a technical restriction that must be specified when the stem cell identity of subpopulations is inferred from their transcriptomic data. Indeed, putative SSC subsets delineated through scRNAseq should be validated by functional assays. However, in the specific case of hSSC transplantation assays, only human spermatogonial colonies could be observed because human germinal differentiation is blocked early in recipient testis of immunodeficient mice [54,96], although it remains the gold standard to test hSSC functionality.

## 6. scRNAseq: A New Way to Explore Spermatogenesis

Because it can measure the expression levels of thousands of genes, scRNAseq supplies a complex but unbiased outlook on tissue differentiation that likely reflects the organization and dynamics of stem and progenitor cells more faithfully than other methods. Thus, scRNAseq unveils the key features of different cell states and transitions and makes it possible to dissect the coordinated but asynchronous ensemble that shapes testicular tissue. Supporting a stage-specific germ cell study at the molecular scale, scRNAseq has proven to be a valuable approach for deciphering stem cell identity. Thus, it revitalizes histology-based analysis of spermatogenesis.

Many scRNAseq experiments have been conducted on murine testicular cells [8,9,10,11,12,13,14,15,16,17,18,19,97]. Green and Wang’s works, both of which were published in 2018, mapped the gene expression dynamics of adult germ cell maturation in mice and humans, respectively [9,19]. They confirmed analogies between mouse and adult human spermatogenesis, thereby validating the mouse as a model for the study of homologous genes that are potentially implicated in human infertility. Hermann et al. also performed parallel processing of RNA from individual spermatogonia of mice and humans [12]. Their results supported the idea that there is a certain maintenance of the transcriptome between mice and humans and suggested that molecular markers of murine germinal cells could be tested to identify clusters among human germ cell lineages, providing support for the exploration of human spermatogenesis but keeping in mind that the mechanisms involved could nevertheless show some differences.

## 7. Resolving Human Spermatogonial Cell Heterogeneity in Discrete Populations with scRNAseq

Many studies have been conducted on human samples at single-cell resolution, providing relevant information on human germinal lineages from fetal germ cells to haploid mature sperm [12,19,20,21,22,23,98,99,100].

In this review, we focus on five recent studies that aimed to characterize the postnatal human germinal lineage at the single-cell level [12,19,20,21,23]. The adult human testicular biopsies that were screened in the experiments originated from 17- to 60-year-old men, living or deceased. In addition to organ donation for research, medical indications for surgical operation were various, ranging from testicular sperm extraction because of obstructive azoospermia to vasectomy reversal surgery. Cells were extracted from fresh [12,19,20] or frozen tissue [21,23]. Various strategies were adopted, ranging from targeted experiments on SSEA4^pos^ cells selected by magnetic-activated cell sorting (MACS) [20] to unbiased approaches that used only whole testicular cells [21] and unbiased studies combined with targeted analyses of ITGA6^pos^ cells, DDX4^pos^ cells, GPR125^pos^ cells or HLA-ABC^neg^CD49e^neg^ITGA6^pos^THY1^dim^EpCAM^dim^ cells selected by FACS or MACS [12,19,23]. Whereas pre-enrichment of spermatogonial populations was expected to make hSSC transcriptome analyses more powerful, selection of DDX4^pos^ or GPR125^pos^ cells proved to be useless as no enrichment in spermatogonia was observed [19], and ITGA6^pos^ cells were found to be contaminated by somatic cells [23]. This selection might even lead to misleading conclusions, as the use of markers, even those considered to be consistent, can prevent isolation of the desired cells. Thus, based on an unbiased analysis, Guo et al. suggested that SSEA4^pos^ cell selection [20] was not relevant to identifying the putative most primitive hSSC subset (see below [21]). The C1 Platform (Fluidigm) or 10× Genomics^®^ technologies were used to conduct RNA sequencing of the sorted cells [20], while either the 10× Genomics^®^ platform [12,21,23] or manual cell picking/FACS followed by modified Smart-seq2 [19] were used in unbiased approaches. The cell number varied greatly as a function of the starting material and the technique used; for example, 64 SSEA4^pos^ cells were analyzed with the C1 platform [20], while the transcriptomes of 6490 cells retained after QC were obtained using the 10× Genomics^®^ platform in an unbiased analysis of total testicular cells [21] (Table 1).

**Figure 2:**
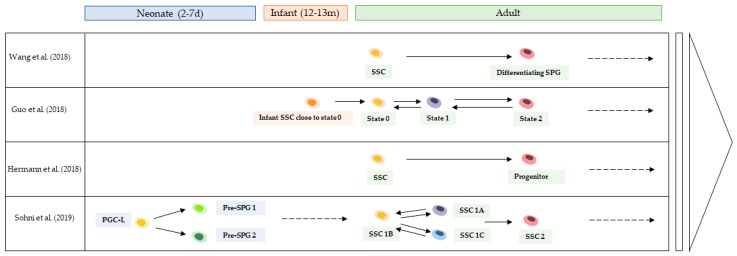
Timeline of hSSC development from birth to adulthood, summarizing the results from the different scRNAseq analyses on human postnatal SSC [12,19,21,23]. Days (d), Human spermatogonial stem cell (hSSC), Months (m), Primordial germ cells like (PGC-L), Single-cell RNA sequencing (scRNAseq), Spermatogonia (SPG), Spermatogonial stem cell (SSC).

After clustering and single-cell trajectory inferences, the human adult spermatogonial population was found to be highly heterogeneous. Three [19], 4 [20,23], 8 [21], and 10 [12] distinct germ cell clusters were distinguished. The developmental trajectories reflecting the transition of hSSC from quiescence to proliferation were traced, and the commitment of hSSC to the differentiation process was divided into 3 [12,19,23], 4 [20], or 5 [21] different cell states (Figure 2). In each of the studies, a spermatogonial cell subset that was regarded as the most primitive and therefore potentially containing hSSC, was defined based on the overexpression of typical hSSC markers such as UTF1, ID4, GFRα1, and FGFR3. By sequencing the transcriptomes of SSEA4^pos^ and cKit^pos^ cells, Guo and colleagues first identified four clusters that they designated “states 1 to 4” [20]. An additional “State 0” was identified based on an unbiased experiment conducted on an unselected whole testicular cell suspension [21]. As a matter of fact, this state was presumed to be excluded from the former experiment as it was associated with low transcription of the *ST3GAL2* gene, which is involved in *SSEA4* expression. Both experiments suggested that State 0 and State 1 may represent two distinct quiescent hSSC states [20,21]. Interestingly, Sohni et al. identified 3 distinct cell states within the SSC-1 subset corresponding to the undifferentiated spermatogonia. Among the 3 sub-clusters (SSC-1A, SSC-1B, and SSC-1C), SSC-1B was regarded as the most primitive and therefore the most enriched in hSSC. In Sohni et al.’s model, SSC-1B cells could convert into SSC-1A or SSC-1C, which represent alternative stem cell states, that would divide into progenitors, that would then be committed to differentiation [23]. In Hermann’s study, the 4 earliest-drafted groups of cells that expressed known spermatogonial genes were refined into 10 spermatogonial clusters with distinctive differentially expressed genes. The identity of human spermatogonial subcategories was inferred from murine transcriptional data, notably through the screening of orthologous gene expression associated with stemness in mice. Interestingly, a novel hSSC subset associated with the hepatic stellate cell activation pathway was identified; it was placed before the pool of typical (i.e., based on the current knowledge) hSSC using pseudotime trajectory analysis. The cells that expressed theoretical known markers of hSSC were found in the middle of the developmental trajectory, suggesting that the identity and the heterogeneity of the hSSC population might be more complex than expected [12].

The germ cell types that appear successively during the first wave of human spermatogenesis differ from those that appear during steady-state adult spermatogenesis, as previously observed in murine models during the first weeks after birth [101]. The characterization and comparison of the expression profiles of human fetal, neonatal and adult germ cells is of interest because it may lead to a description of the development of the testis throughout life and of the regulatory mechanisms that govern cell fate. It should also contribute to fertility preservation in young boys via the identification and maturation of prepubertal hSSC. Tracing the ontogenesis of postnatal hSSC using a single-cell high-throughput (10× Genomics^®^) approach was initiated by Sohni et al., who analysed all testicular cells (14,862) from the testes of two-day-old and seven-day-old newborns [23]. This unbiased approach revealed the existence of 2 neonatal germ cell clusters, one of which displayed an expression profile highly reminiscent of that of primordial germ cells (PGC) in fetal life, as reported by Guo et al. in 2015 [98] (so-called “PGC like” (PGCL)); the second cluster, which exhibited a transcript pattern similar to that of adult hSSC, was designated as “prespermatogonia” (PreSPGs) and was itself delineated into 2 distinct groups—“PreSPG-1′’ and ‘‘PreSPG-2′’ [23]. These results suggest that neonatal PGCL derived from fetal PGC give rise to PreSPGs (Figure 2). In their study, Guo et al. explored the unbiased single-cell transcriptome of testes from 2 deceased boys (ages 12 months and 13 months) [21]. They noted that gene expression in infant germ cells and adult State 0 cells was similar and positioned the infant germ cells at the beginning of the developmental trajectory, shortly forward of State 0, in agreement with the suggested pattern of differentiation of germ cells into spermatogonia that occurs in the first year of human life [102]. This population of infant germ cells should represent the quiescent “reserve” stem cell pool that exists from the first year of life until puberty.

As the transcriptomic exploration of human germ cells progresses, one may hope to reconstruct the entire developmental process of spermatogenesis from in utero to adulthood. A substantial lack of data covering the period from 1–13 years of age in the biological timeline is noticeable (Figure 2), and this will be undoubtedly rapidly filled by future analyses.

## 8. Stem Cell Hierarchy: A Revisited View of Stem Cell Paradigm

In the human pre-meiotic phase, quiescent A_dark_ and actively dividing A_pale_ spermatogonia are considered to represent stem cell pools that will then commit to development into B differentiating spermatogonia. However, the biological relevance of this separation of hSSC into 2 groups was recently challenged based on immunostaining and transcriptomic data that highlighted the many similarities between A_pale_ and A_dark_ spermatogonial subpopulations but also the molecular diversity within each of these histologically defined groups [103,104]. Different spermatogonial subtypes can coexist in A_dark_ and A_pale_ populations, suggesting that hSSC classification based on nuclear morphology could be obsolete. Consistent with this, single-cell transcriptomics studies confirmed that the primitive spermatogonial human population containing A_dark_ and A_pale_ spermatogonia is highly heterogeneous. For example, in Hermann’s study [12], up to 10 distinct cell clusters were found to describe the transition from A_dark_, A_pale_ to B spermatogonia. In addition, 3 distinct cell states were identified within the undifferentiated SPG population in Sohni’s study [23], and 2 distinct quiescent SSC states were delineated in Guo’s work [21].

Hence, recent work in the field of scRNAseq confirmed that the division of the primitive hSSC population into A_dark_ and A_pale_ spermatogonia needs to be revisited; this exemplifies the great contribution of single-cell omics approaches to defining new models. However, the concept of reserve and active groups of stem cells, together with the occurrence of exchanges between the 2 pools of cells, remains very attractive. By integrating all germinal cells in a transcriptional panorama, scRNAseq data challenges the paradigm of clearly delimited stem and progenitor cell types, as these data can unveil some subpopulations and blur the delineations between cell stages [105]. In most of the analyses of human testicular single cells mentioned above, the developmental trajectories observed support the linear model of stem cell hierarchy. However, Sohni et al., who conducted a more detailed study of undifferentiated spermatogonia, reported a more complex trajectory pattern, i.e., a non-linear hSSC differentiation process [23] with 2 alternative differentiation pathways for the most primitive hSSC subset. Interestingly, Guo’s team inferred the dynamics of the developmental trajectories in States 0, 1, and 2 via an RNA velocity approach. RNA velocity represents the time derivative of the gene expression state and is calculated from the amounts of nascent (unspliced) and mature (spliced) mRNA in individual cells. This vector allows prediction of the evolution of gene expression and the future state of individual cells on a timescale [106]. Thus, Guo et al. observed trends for forward and backward trajectory movements between the 3 states of interest. This phenomenon suggested that the cells could be in different interconvertible states, revealing a certain plasticity of hSSC and spermatogonia pools [21]. The fact that open chromatin and the DNAme landscape in SSEA4^pos^ cells and c-KIT^pos^ spermatogonia did not vary much may shed light on the potential transition and reversion between hSSC and spermatogonial states [20]. This metastable behavior is reminiscent of murine models in which some spermatogonia of different states can interconvert [40,107]. This alternative model grants hSSC and progenitor cells a better capacity to adapt to changes, enabling the maintenance of niche homeostasis, notably in case of injury. This is consistent with the proposals of Clermont, who suggested that different types of stem cells might coexist as reserve and proliferating cells, with the latter able to replenish the former in primates [24].

## 9. Identification of the Most Putative SSC and Molecular Pathways Regulating Their Maintenance

The use of scRNAseq data to infer hSSC markers and to define the most likely hSSC population presents another major challenge. Guo et al. defined the most primitive hSSC state using surface markers such as FGFR3^high^, TSPAN33^high^, and SSEA4^low^; the TSPAN33 marker was also used in Herman’s and Sohni’s works [12,21,23]. In addition, Guo and colleagues placed the UTF1^high^ GFRα1^low^ hSSC subset at the beginning of the developmental trajectory; loss of UTF1 expression and gain of GFRα1 expression were associated with spermatogonial differentiation, in agreement with the studies of Wang et al. [19]. However, this hSSC phenotype contradicts the conclusions of Di Persio and colleagues, who suggested that primitive hSSC are GFRα1^high^UTF1^neg^ [108]. Sohni et al. challenged the status of GFRα1, FGFR3, and UTF1 as reliable specific hSSC markers because GFRα1, FGFR, and UTF1 expression was observed across different hSSC subsets and even in differentiating spermatogonia [23]. In addition to TSPAN33, they identified PIWIL4 and LPPR3 as candidate markers; the PIWIL4 protein proved to be a more specific marker than UTF1, in agreement with previous results showing that PIWIL4 is expressed in a subpopulation of FGFR3^high^ spermatogonia [19] or by cells co-expressing UTF1 and GFRα1 [20]. Although invaluable insights into hSSC identity are provided by the scRNAseq approach, additional work is needed to reconcile the discrepancies in these data. This would lead to the identification of a combination of cell markers that identify the most likely hSSC subset. Another layer of complexity could also arise when the stemness of these candidate populations will be checked by functional tests such as transplantation to the testis of immunodeficient mice, resulting in the classification of these subpopulations in terms of their long-term regenerative potential in the recipient testis. As a matter of fact, while SSEA4^pos^ might not be expressed in the earlier stem cell state [21], SSEA4^pos^ hSSC were shown to be able to regenerate colonies of spermatogonia, at least in the short term, i.e., 4 weeks after transplantation [109].

Based on compelling evidence showing cell-cycle gene expression patterns, the scRNAseq studies mentioned above confirmed that the most undifferentiated hSSC subset is quiescent, consistent with the lack of expression of the proliferation marker KI67 [21]. The preferential expression of genes involved in mitochondrial function and oxidative phosphorylation at the end of developmental trajectories tended to show that transition from quiescence to a differentiated state is supported by a metabolic shift from glycolysis to oxidative phosphorylation [12,20]. Interestingly, upregulation of GDNF, FGF2, and WNT signaling pathways was also observed in the hSSC subset [19,20]. These molecular pathways play a critical role in regulation of the self-renewal and maintenance of mSSC [40,47,50]. In addition, EIF2, mTOR, and p70S6K signaling were preferentially expressed in the hSSC subset, suggesting the potential ability of these signaling pathways to control the translation process in hSSC [12].

## 10. Unbiased scRNAseq of Whole Testis: A Tool to Describe the Testicular Niche

In addition to germ line characterization, scRNAseq explores the interactions between the cell subpopulations that form testicular tissue, including the specialized somatic cells that provide the requisite support to SSC and affect germ cell development. The molecular features and regulatory networks that create this environment remain to be brought to light. In view of this, unbiased scRNAseq experiments on testicular cells have provided a wealth of information on the testicular niches of mice [8,9,10], human neonates [23], infants [21], fertile men and men with obstructive azoospermia [21,23,91]. In these studies, the major testicular somatic cell types were delineated according to the expression of several marker genes. Wang’s team distinguished 4 somatic cell types—SC, macrophages, PMC and LC (the latter two were first clustered together) [19]. Sohni et al. enumerated 4 groups of cells in neonatal testis (SC, PMC, LC, and endothelial cells) and 3 in adult testis (PMC, LC, and endothelial cells) [23], while Guo’s team found 5 clusters (SC, PMC, LC, endothelial cells, and macrophages) [21], as was also found by Green et al. in adult mice [9]. Green and colleagues discovered 2 other somatic cell types within the murine testis: an innate lymphoid type II and an unknown mesenchymal cell type. This study also focused on SC that displayed significant heterogeneity; among the 4 types of SC, 9 subtypes were distinguished and spatially defined according to the stages of the seminiferous epithelial cycle [9]. Although they require further investigation, these breakthroughs signify a great advance in the understanding of the composition and interactions within the testicular microenvironment. SC in the human niche might also prove to be less invariable than was previously thought. However, notable differences between the two species with respect to the intercellular relationships within the niche were observed by Guo et al. [21]. They also reported that the 40-μm filtering step limits the capture of large SC. Likewise, SC were lacking in Sohni et al.’s study due to their size [23]. Sohni et al. noted changes in the gene expression of somatic cells in the neonatal and adult testis and highlighted developmental shifts that occur in LC and PMC. Perseverance in exploring the somatic cells that form the niche is nevertheless essential as it could offer a novel comprehensive view of the crosstalk mechanisms and regulatory interactions that occur within the testicular microenvironment and control its ontogenesis. scRNAseq holds great promise for understanding and retracing the implementation of this complex microenvironment, paving the way for its clinical application in reproduction in the future.

## 11. Current Challenges and Prospects for Infertility Diagnosis

Transcriptomic analysis will lead to the establishment of a testicular tissue gene expression atlas that is qualitative as well as quantitative and provides key information that can be used in the diagnosis and investigation of male infertility. ScRNAseq offers advantages compared to classical anatomical pathology analyses and bulk sequencing approaches because it can not only highlight cell subtypes that may be missing from a testis biopsy but can also assess transcriptional changes that may lead to impaired spermatogenesis.

First, scRNAseq can be used to identify unusual gene expression patterns or differences in testicular tissue cell composition that are associated with known infertility pathologies such as chromosomal (Klinefelter syndrome; chromosome translocations, inversions, and deletions) or genetic defects (microdeletions in the Y chromosome azoospermia factor region or mutations in the androgen receptor gene, for example) [110,111,112]. Second, the datasets provided by single-cell transcriptomics could complete knowledge in these cases of impaired spermatogenesis, outlining mechanisms that are indiscernible through other approaches.

Although many genetic mouse models of infertility have been described [113,114], few genetic causes of infertility have so far been identified in humans, in part as a result of a poor understanding of the basic regulatory mechanisms. ScRNAseq could contribute to the resolution of diagnoses by permitting a comparison of transcriptomic data from patients with arrested or disturbed spermatogenesis with transcriptomic data from fertile patients, which could be taken as a reference. This should support a finer classification of male infertility defects, as genetic variations, regulatory elements, and phenotype could be linked. Thus, the elucidation of the causes of infertilities that as yet are unexplained and are currently defined as idiopathic can be expected. For this purpose, Wang’s team screened 174 testicular cells from testis of a man with non-obstructive azoospermia (Sertoli cell–only syndrome). ScRNAseq identified all the cells as somatic (SC, PMC, or Leydig cells),confirmed previous anatomo-pathological examinations and extended their identification. The comparison of datasets from a patient with non-obstructive azoospermia with datasets from donors with normal spermatogenesis permitted the identification of many differentially expressed genes of each somatic cell type that could be involved in gametogenesis, as discussed by the authors [19]. In the same vein, Hermann and colleagues measured homologous gene expression in spermatocytes from mice and humans; in mice, ablation of these genes is associated with maturation arrest in spermatogenesis [12]. These first results are very encouraging and suggest that, in the future, it may be possible to find out new molecular pathways, whose dysregulation could be potentially involved in human infertility. Their implication should be further studied and validated in animal models.

Through this highlight of dysregulated pathways in specific testicular cell types, it might be assumed that scRNAseq will support personalized reproductive medicine as a useful provider of biomarkers for diagnostic. For example, the transcriptomic snapshot of the different cell compartments of a patient’s testicular biopsy could help identify the somatic and/or germinal origin of the spermatogenic defect, leading to an appropriate genetic counseling.

## 12. Single-Cell Omics: New Tools for Translational and Clinical Research in the Field of Reproduction

While genomics measures the sequences of genes, transcriptomics, proteomics, and metabolomics display the dynamics of their products. Multi-omics single-cell data could hence improve our understanding of human steady-state spermatogenesis and thus make it possible to address the causes of disrupted germinal cell differentiation. Various single-cell approaches to the study of the differentiation process should complement one another and highlight the molecular pathways that regulate cell states and transitions. Pairing data from proteomics and transcriptomics could yield further insights by linking signaling and regulatory proteins with mRNA expression, considering that discrepancies can occur owing to miRNA control of translation or to post-translational regulation at the protein level, for example. New tools have been developed that can be used to sequence the genome and transcriptome of the same cell in parallel, making it possible to compare data and to enrich each analysis. This will help investigators find mutations and transcriptional variations that are correlated with impaired spermatogenesis [115,116]. The combined use of epigenomic and transcriptomic data may also contribute to our understanding of regulatory pathways, considering the importance of epigenetics in gene regulation [117]. Methods that provide insights into chromatin accessibility and genetic regulatory elements have been developed, including whole genome bisulfite sequencing (WGBS), chromatin immunoprecipitation sequencing (ChIP-seq), and an assay for transposase-accessible chromatin with high-throughput sequencing (ATAC-seq) [118]. The identification of open chromatin patterns has been widely used to map candidate regulatory sequences. This is an important step toward deciphering the molecular pathways involved in the regulation of stem cell maintenance and differentiation. In this vein, Guo and colleagues combined single-cell transcriptomic data with dynamic chromatin states obtained via bulk ATACseq and WGBS analyses to document the signaling and metabolic regulation that occurs during spermatogenesis [20]. Epigenomic profiling has recently been extended to single-cell resolution and coupled with scRNAseq [119]. We can speculate that this strategy will be used to explore testicular tissue in the coming years. Pairing of scRNAseq data with scATAC-seq data has already been used to explore lineage dynamics in human hematopoietic tissue [120]. However, these methodologies screen the epigenome with low coverage, especially when only a few thousands of cells are studied. Nonetheless, technical overcoming of this current limitation can be expected. The great advances that have occurred in recent years lead us to anticipate that an exhaustive description of SSC based on all aspects of omics will be available in the near future, permitting the unravelling of various issues related to the molecular mechanisms that regulate germ cell fate.

Because they offer characterization of hSSC and highlight the genetic, epigenetic and environmental factors that govern the proliferation and differentiation of hSSC, scRNAseq, and other single-cell omics appear to be precious tools for the development of potential future therapies. Kyle Orwig′s team demonstrated that transplantation of autologous and allogeneic SSC into rhesus macaque testis can result in the production of functional sperm [34]. A similar protocol could be applied to humans in clinical practice as a restoration strategy in cases of gonadotoxic agent–induced azoospermia. Especially in prepubertal cases, in vitro SSC amplification is essential to obtain enough SSC to transplant and thereby achieve efficient recovery of spermatogenesis in the recipient testis. Deciphering the molecular pathways that initiate and support SSC expansion in vitro is thus critical for this scientific advancement, which is already feasible in various animal models [121]. scRNAseq could help define specific cocktails of growth factors/cytokines that promote in vitro stem cell amplification. Furthermore, scRNAseq describing all steps of spermatogenesis could assist in achieving the sequential steps of differentiation in vitro that lead to haploid germ cells. Sperm derivation from SSC, pluripotent embryonic stem cells (ES), or induced pluripotent stem cells (IPS) in combination with intracytoplasmic sperm injection (ICSI) is a potential therapeutic approach that single-cell omics data could fuel. This has been partially accomplished in humans but has been shown to be efficient in several species, as in the formation of functional male spermatid-like cells from mouse embryonic stem cells (ESC) [122,123,124,125,126,127]. Likewise, scRNAseq data could help in the development of protocols like those that have already been described in mouse models for sperm derivation from in vitro organotypic cultures of human testicular biopsies or culture of testicular organ-like structures called organoids [128,129,130,131,132].

The already productive work on spatial transcriptomics foreshadows the coming ability to draw a spatiotemporal map of the interactions of neighboring cells—stem cells, progenitors and also somatic cells—in the testicular niche. Remarkably, combining scRNAseq with methods of this type led to the creation of a spatially resolved cell atlas of gene expression in the mouse hypothalamic preoptic region [88]. Due to the anatomical preservation of the niche, a similar experiment could not only unveil the real architecture of the testicular microenvironment, which is currently a subject of debate (facultative/open or discrete) in mammals [50] and has not been elucidated in primates, but might also provide valuable information for the development of hSSC based cell therapy.

Moreover, scRNAseq could be of interest as a QC tool for the validation and follow-up of various protocols involving the use of germ cell differentiation in future. We can imagine the implementation of cell therapies using SSC in cases of hypofertility or, in cases of more severe infertility phenotypes (Sertoli cell–only syndrome), the use of SSC-like stem cells or PGCL cells derived from pluripotent ES/IPS cells in vitro [133,134,135,136,137,138], keeping in mind potential epigenetic variability in derived germ cells. If this is done, scRNAseq analysis could also be useful in controlling the functionality of the testicular microenvironment in the recipient testis of the patient prior to treatment to ensure that the niche can properly shelter and support the homing, self-renewal, and differentiation of transplanted SSC. In addition, in genetic therapy or cell therapy of deficient testicular somatic microenvironments, single-cell omics such as scRNAseq could also be applied to control the functionality of the corrected testicular niche. Recently, autografts of cryopreserved prepubertal testicular tissues from rhesus macaques were shown to mature and to produce functional sperm [139], providing new hope for the treatment of infertility in patients after childhood sterilizing treatment. The cell type–specific gene expression signatures provided by single-cell transcriptome studies should also make it possible to control the developmental progression of testicular biopsy grafts obtained from prepubertal and adult patients.

## 13. Conclusions and Perspectives

In the context of spermatogenesis, single-cell transcriptomic analysis has already revealed the presence of somatic and germ cell subtypes that have to date been indistinguishable and unveiled the heterogeneity of SSC. This should result in a finer characterization of spermatogonial populations and a better hierarchization of successive cell states derived from the reconstruction of developmental lineages. It should be noted, however, that the putative defined SSC populations need to be validated through functional assays. Further improvements can, nevertheless, be expected. A combination of several new biomolecular and computational strategies could be used to address the lack of spatial information. In addition, the reconciliation of diverse omics studies on spermatogenesis is another path to profitable knowledge growth. In this regard, the ReproGenomics Viewer (RGV) was set up in 2015. This web-based resource of harmonized cross-species and cross-technology sequencing datasets related to reproduction has been redesigned since then and includes single-cell resolution studies. The creators of the platform now intend to integrate other technologies such as proteomics and bisulfite sequencing experiments into the resource [140]. The combination of emergent approaches and sharing of knowledge will undoubtedly offer additional insights into the stem cell-fate process. Thus, scRNAseq appears to be a promising tool not only for personalized male infertility diagnosis but also for the development of SSC-based therapies in reproductive biology.

## Figures and Tables

**Table 1:** scRNAseq datasets on human spermatogonia (SPG).

Publication	_SC_Rnaseq Method	Selected/Unselected Population	Pathology	Age (Years Old)	Testicular Cell Number	SPG Cluster	Validation Method
**Guo et al. (2017)**	Fluidigm C1	Sorted (MACS): SSEA4^pos^ or c-KIT^pos^	Patient experiencing idiopathic pain, not involving cancer or major inflammation	Five adults (unspecified age)	92 60 SSEA4^pos^ 32 c-KIT^pos^	4 CL: State 1 SSC, state 2, and state 3 differentiating SPG, state 4 differentiated SPG	IHC
**Wang et al. (2018)**	Manual picking Smart-seq2	Unsorted and sorted (FACS): GPR125^pos^/DDX4^pos^	F, OA, NOA	F: 30, 60 OA: 39, 43, 27, 34, 44, 41, 29 NOA: 24	F and OA: 2854 NOA: 174	3 CL: SSC, differentiating SPG, differentiated SPG	ISH and IHC
**Guo et al. (2018)**	10× Genomics	Unsorted and sorted (MACS): c-KIT^pos^	Deceased patients without testicular pathology	Young adults: 17, 24, 25 Infants: 12 and 13 months	Young adults: 6490 Infants: 1300	Five CL for adults: States 0, 1, and 2: SSC and most primitive SPG, states 3, 4: differentiating SPG One CL for infants: SSC “state 0”	seqFISH
**Hermann et al. (2018)**	Fluidigm C1 and 10× Genomics	Unsorted and sorted: HLA-ABC^neg^/ CD49e^neg^/Thy1^dim^/ ITGA6^pos^/EpCAM^dim^	Patient undergoing microscopic vasectomy reversal, OA and organ donor	Adults (C1): 50, 40, 38, 46, 35, 54, 53, 30, 40 Adults (10×): 37, 38, 34, 36, 49, 43, 43	Unsorted: 7134 Sorted spg:11104 (10×) + 635 (C1)	Four CL (further subdivided in 10 subclusters): Two undifferentiated SPG, and differentiated SPG	IHC and RT-qPCR
**Sohni et al. (2019)**	10× Genomics	Unsorted and sorted (MACS): ITGA6^pos^	Patient undergoing vasectomy reversal	Adults: 37, 42 Neonates: 2 and 7 days	Adults: 18,723 (7974 sorted) Neonates: 14,862 (6086 sorted)	4 CL: SSC1 (1B; 1A; 1C); SSC2; early differentiating SPG; differentiating SPG	IHC

Clusters (CL), Fertile patients (F), Fluorescence-activated cell sorting (FACS), Immunohistochemistry (IHC), Magnetic-activated cell sorting (MACS), Non-obstructive azoospermia (NOA), Obstructive azoospermia (OA), Reverse transcriptase quantitative polymerase chain reaction (RT qPCR), RNA in situ hybridation (ISH), Sequential fluorescence in situ hybridization (seqFISH), Single-cell RNA sequencing (scRNAseq), Spermatogonia (SPG), Spermatogonial stem cell (SSC).
